# Heat-Killed *Trypanosoma cruzi* Induces Acute Cardiac Damage and Polyantigenic Autoimmunity

**DOI:** 10.1371/journal.pone.0014571

**Published:** 2011-01-21

**Authors:** Kevin M. Bonney, Joann M. Taylor, Melvin D. Daniels, Conrad L. Epting, David M. Engman

**Affiliations:** 1 Department of Pathology, Northwestern University, Chicago, Illinois, United States of America; 2 Department of Microbiology-Immunology, Northwestern University, Chicago, Illinois, United States of America; 3 Department of Pediatrics, Northwestern University, Chicago, Illinois, United States of America; New York University, United States of America

## Abstract

Chagas heart disease, caused by the protozoan parasite *Trypanosoma cruzi*, is a potentially fatal cardiomyopathy often associated with cardiac autoimmunity. *T. cruzi* infection induces the development of autoimmunity to a number of antigens via molecular mimicry and other mechanisms, but the genesis and pathogenic potential of this autoimmune response has not been fully elucidated. To determine whether exposure to *T. cruzi* antigens alone in the absence of active infection is sufficient to induce autoimmunity, we immunized A/J mice with heat-killed *T. cruzi* (HKTC) emulsified in complete Freund's adjuvant, and compared the resulting immune response to that induced by infection with live *T. cruzi*. We found that HKTC immunization is capable of inducing acute cardiac damage, as evidenced by elevated serum cardiac troponin I, and that this damage is associated with the generation of polyantigenic humoral and cell-mediated autoimmunity with similar antigen specificity to that induced by infection with *T. cruzi*. However, while significant and preferential production of Th1 and Th17-associated cytokines, accompanied by myocarditis, develops in *T. cruzi*-infected mice, HKTC-immunized mice produce lower levels of these cytokines, do not develop Th1-skewed immunity, and lack tissue inflammation. These results demonstrate that exposure to parasite antigen alone is sufficient to induce autoimmunity and cardiac damage, yet additional immune factors, including a dominant Th1/Th17 immune response, are likely required to induce cardiac inflammation.

## Introduction

Chagas disease, along with African sleeping sickness and leishmaniasis, is one of a triumvirate of diseases caused by protozoan parasites belonging to the family *Trypanosomatidae*. Chagas disease results from infection with *Trypanosoma cruzi,* which is endemic to Central and South America [1]. Acutely, some patients develop fatal myocarditis, some clear the infection with minimal symptoms, and some develop a chronic infection leading to an inflammatory cardiomyopathy termed Chagas heart disease (CHD) [2]. Cardiac autoimmunity is among many contributing factors proposed to explain the variable course and outcome of CHD [3], [4], [5], [6], [7], [8], [9], [10], [11], [12], [13], [14], [15], [16]. Although it is likely that a combination of distinct mechanisms drives CHD pathogenesis, the role of autoimmunity is of specific interest to our laboratory to inform the design and application of new therapies.

The presence of cardiac autoimmunity is well documented in mice and humans infected with *T. cruzi* [2], [7], [17], [18], [19], [20]. There is an ongoing debate regarding autoimmunity in CHD centered on two major questions: By what mechanism(s) is autoimmunity induced, and what role does autoimmunity play in disease pathogenesis? CHD-related autoimmunity may be initiated by parasite-induced damage to cardiomyocytes and/or by molecular mimicry between immunologically similar epitopes of parasite and host proteins. Cardiac myosin has been identified as a prominent autoantigen in a variety of cardiac infectious/autoimmune diseases, including CHD [5], [18], [21], [22]. In models of experimental autoimmune myocarditis (EAM), immunization with cardiac myosin, recombinant myosin fragments, or myosin peptides induces cardiac inflammation [23], [24]. Even though a robust autoimmune response against cardiac myosin is detected during *T. cruzi* infection, our lab has shown through induction of antigen-specific T-cell tolerance that myosin autoimmunity is not essential for cardiac inflammation in acute CHD [25]. However, using a different strategy of immunologic tolerance induction, Pontes-de-Carvalho and colleagues were able to successfully prevent autoimmune myocarditis in mice chronically infected with *T. cruzi* by tolerizing with an emulsion of cardiac homogenate containing myosin, actin, and numerous other unidentified cardiac proteins [26]. Together these results indicate that autoimmunity may contribute to CHD pathogenesis, but that autoreactive immune responses to proteins other than myosin are required for the induction of autoimmune myocarditis in experimental CHD. We have previously demonstrated that immunization with *T. cruzi* protein extract in CFA induced cross-reactive humoral and cellular autoimmunity against cardiac myosin, but did not induce myocarditis [27]. This suggested that while molecular mimicry between *T. cruzi* antigens and cardiac myosin occurs, *T. cruzi* antigen-induced autoimmunity may not be sufficient to drive myocarditis, although the mechanisms underlying this disconnect were not apparent. It remains unclear how immunization with myosin or myosin fragments initiates autoimmunity with associated inflammation, yet immunization with *T. cruzi* antigens drives only autoimmunity. Importantly, the spectrum and type of the cellular immune response was not thoroughly examined in previous studies, leaving important unanswered questions regarding the role autoimmunity might play in CHD pathogenesis.

Immunity to cardiac autoantigens other than myosin have been identified in *T. cruzi*-infected humans and in animal models of CHD [5], [28], [29], [30], [31], [32], but a comprehensive study of this polyantigenic autoimmunity in a single model system has not been described. Here, we tracked the development of autoimmunity against a panel of known autoantigens in a single model system, comparing the autoimmunity resulting from *T. cruzi* infection and immunization with heat-killed *T. cruzi* (HKTC). This approach allowed us to confirm the hypothesis that HKTC immunization can induce autoreactive responses to antigens other than myosin, and to analyze qualitative and quantitative differences in the type and magnitude of humoral and cellular autoimmunity induced by exposure to *T. cruzi* antigens or to an active infection. Specifically, we assessed Th1 and Th17 responses in *T. cruzi*-infected and HKTC-immunized mice, since Th1 cells are important for clearing *T. cruzi* infection and both Th1 and Th17 cells are known to drive the development of a number of autoimmune diseases [23], [33], [34], [35], [36], [37], [38]. We also employed a more sensitive assay to measure cardiac injury than had been used previously in experiments involving *T. cruzi* lysate immunization, Because immunization with *T. cruzi* proteins may induce cardiac injury that is of physiological relevance despite the absence of observable cardiac inflammation, we employed a sensitive assay to assess cardiac injury by measuring cardiac troponin I (cTnI) [39], [40].

## Results

### Immunization with heat-killed *T. cruzi* induces acute cardiac damage without myocarditis

**Figure 1:**
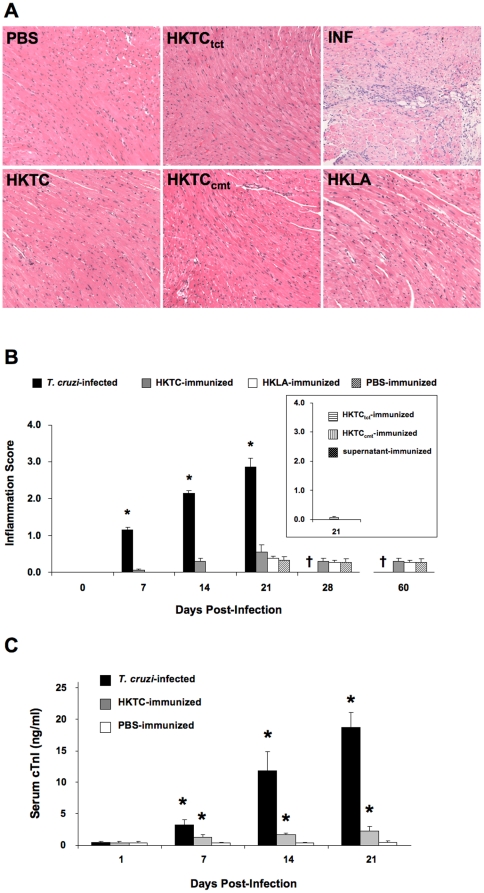
Immunization with heat-killed *T. cruzi* causes acute cardiac damage but not myocarditis. A/J mice were infected with *T. cruzi*, immunized with heat-killed *T. cruzi* epimatigotes (HKTC), *T. cruzi* tissue-culture trypomastigotes (HKTC_tct_) *T. cruzi* cultured metacyclic trypomastigotes (HKTC_cmt_) or injected with PBS. (A) One representative image from each group at 21 d.p.i. is shown. (B) The mean histopathology score for inflammation is indicated. For 7, 14, and 21 d.p.i., n = 10 for all groups; for 28 d.p.i. n = 5 for HKTC-immunized, heat-killed *L. amazonensis* (HKLA)-immunized, and PBS-immunized groups. (C) The mean serum cTnI level is indicated, n = 4 for *T. cruzi*-infected and PBS-immunized groups; n = 6 for the HKTC-immunized group. (B and C) Error bars indicate SEM. * *P*<0.05 compared to the PBS control group. † *T. cruzi*-infected mice were not analyzed at this time point because they do not survive past 25 d.p.i.

Infection of A/J mice with *T. cruzi* Brazil strain results in the development of inflammatory myocarditis characterized by mononuclear cell infiltration, edema, and myocyte degeneration. We compared myocardial histology from mice infected with parasites to those immunized with heat-killed *T. cruzi* epimastigotes (HKTC) at days 7, 14, and 21 post infection/immunization, and assessed HKTC-immunized mice for signs of histological disease at days 28 and 60 days post-immunization. Similar heat-killed preparations of *L. amazonensis* (HKLA), a related organism with many shared antigens, yet not known to cause cardiac pathology, were used as a negative control to demonstrate specificity of the response. Because the protein expression pattern differs among *T. cruzi* life cycle stages, we also immunized mice with HKTC made from tissue culture-derived trypomastigotes (HKTC_tct_) and *in vitro* cultured-derived metacyclic trypomastigotes (HKTC_cmt_) to verify that immunization with these more clinically-relevant life cycle forms produced a similar results (Fig. 1A and B). To address the possibility that contaminating antigens from the rat cardiac myocytes used to propagate the *T. cruzi* trypomastigotes was contributing to the resulting autoimmunity and inflammation, we immunized control mice with parasite-cleared supernatants of from infected cultured H9C2 myocytes (Fig. 1B). We found that only *T. cruzi*-infected mice developed significant myocarditis, characterized by mononuclear cell infiltration, myocyte necrosis, and edema (Fig. 1A), while HKTC-immunized, HKLA-immunized, and PBS-immunized mice did not develop signs of myocarditis through 60 d.p.i., and HKTC_tct_-immunized, HKTC_cmt_-immunized, and supernatant-immunized mice did not show signs of cardiac inflammation when assessed at 21 d.p.i. (Fig. 1A and B). The average inflammatory response was quantified over time (Fig. 1B), revealing that *T. cruzi*-infected mice developed progressive myocarditis from day 7 onward, with death occurring around day 25.

We next assessed acute cardiomyocyte damage, as measured by serum cardiac troponin I (cTnI) levels. Elevated cTnI levels were detectable in infected mice as early as 7 d.p.i. (Fig. 1C) and steadily increased through day 21 (Fig. 1C). Importantly, immunization with HKTC also resulted in a statistically significant elevation of cTnI by 7 d.p.i., which persisted through 21 d.p.i. (Fig. 1C), when compared to PBS-immunized controls.

### HKTC-immunized mice develop humoral autoimmunity with similar antigen specificity as *T. cruzi*-infected mice

**Figure 2:**
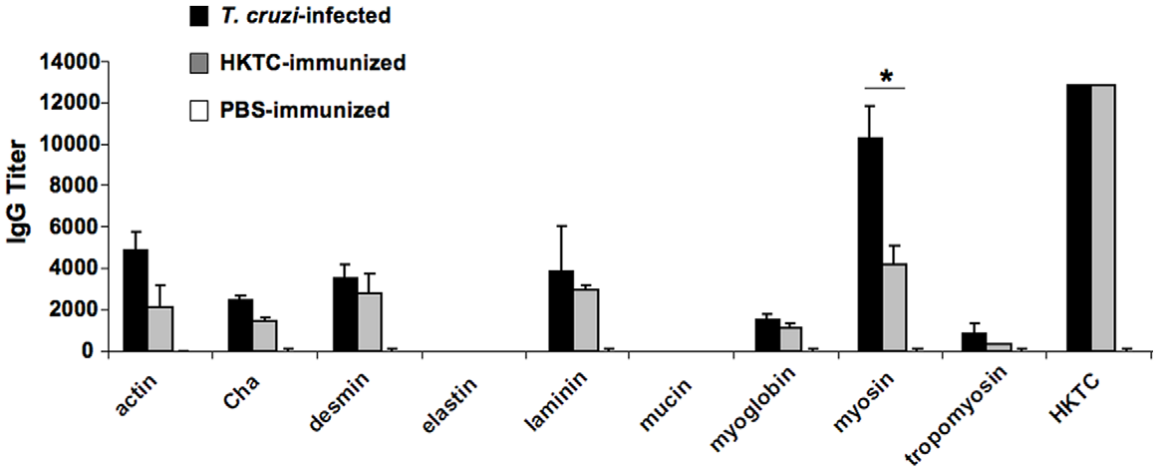
Development of humoral autoimmunity in mice immunized with HKTC is of similar antigen specificity as in *T. cruzi* infected mice. A/J mice were infected with *T. cruzi* (n = 7), immunized with HKTC (n = 7), or injected with PBS (n = 3). ELISA analysis was used to test individual serum samples for IgG reactivity to *T. cruzi* as well as to a panel of cardiac antigens (actin, Cha antigen, desmin, laminin, myoglobin, myosin, tropomyosin) and non-cardiac antigens (elastin, mucin) or the immunologically irrelevant antigen BSA at 21 d.p.i. Each data point represents the mean antibody titer of each cohort above the background response to BSA. Error bars indicate SEM. Data are representative of two independent experiments. * *P*<0.05.

*T. cruzi*-infected mice develop polyantigenic humoral reactivity, therefore we hypothesized that HKTC immunization would induce humoral autoimmunity to a number of autoantigens in addition to myosin. We examined total serum reactivity against whole heart homogenates by immunoblot, and found that both *T. cruzi*-infected and HKTC-immunized mice developed immunoreactivity to an array of cardiac proteins by 21 d.p.i. (data not shown). To obtain a quantitative measure of antibody production and track the temporal development of humoral autoimmunity against a panel of known cardiac autoantigens, we performed ELISAs comparing sera from *T. cruzi*–infected and HKTC-immunized mice (Table 1). Although there was a slight delay in the development of some of the autoantibody responses in HKTC-immunized mice (data not shown), by 21 d.p.i. both groups of mice developed significant autoreactivity against antigens abundant in the heart: actin, Cha antigen, desmin, laminin, myoglobin, myosin, and tropomyosin (Fig. 2). There was no detectable response against lung-derived elastin or stomach-derived mucin in any of the groups (Fig. 2), suggesting a cardiac specificity to the autoimmunity. There was no significant difference in the antibody titers between these two groups for any of the antigens except for myosin. As expected, both groups mounted a significant response against HKTC (Fig. 2), and there were no significant autoantibody responses in the HKLA-immunized mice (data not shown).

**Table 1:** A number of cardiac proteins have been identified as targets of autoimmunity in humans patients with and experimental models of Chagas heart disease.

Protein	Immunological relevance
Actin	autoantibodies induced in *T. cruzi*-infected mice^a^
Cha	reactivity from human Chagasic sera and from sera and T cells in *T. cruzi*-infected mice^b^
Desmin	autoantibodies induced in *T. cruzi*-infected mice^c^
Laminin	reactivity from human Chagasic sera and from sera and T cells in *T. cruzi*-infected mice^d^
Myoglobin	autoantibodies induced in *T. cruzi*-infected mice^c^
Myosin	autoantibodies and T cell responses in *T. cruzi*-infected mice^b^,^c^,^e^
Tropomyosin	autoantibodies induced in human patients

a BALB/c x C57Bl/6,

b C57Bl/6,

c CBA/J,

d BALB/c,

e A/J.

### The IgG2a/IgG1 ratio of the antibody response against a panel of cardiac antigens is higher in *T. cruzi*-infected mice than in HKTC-immunized mice

To assess qualitative differences in humoral autoimmunity between *T. cruzi*-infected and HKTC-immunized mice, we examined the IgG isotypes of antigen-specific antibodies in the two groups, and compared these responses to those of PBS-injected control mice. Of note, the total IgG titers were similar, however, we focused on IgG1 and IgG2a because these two isotypes account for 90% of the IgG produced in Chagas patients, and are most associated with differential immune responses; IgG1 is associated with a dominant Th2 response and IgG2a is associated with a dominant Th1 response [41]. Using ELISA, we determined that both *T. cruzi*-infected and HKTC-immunized mice developed significant IgG1 and IgG2a responses to most of the antigens tested (actin, Cha antigen, desmin, laminin, myoglobin, myosin, tropomyosin, and HKTC) which the PBS-immunized control animals lacked (Table 2). In general, the infected animals had significantly greater titers of both IgG2a and IgG1 than did HKTC-immunized mice (Fig. 2). We next calculated the IgG2a/IgG1 ratio of the autoantibody responses to determine whether HKTC immunization recapitulated the IgG isotype profile induced by *T. cruzi* infection, and discovered a striking difference between infected and immunized animals. The infected animals demonstrated an IgG response skewed toward IgG2a to 5 out of 7 autoantigens as well as against HKTC, while HKTC-immunized animals developed a dominant IgG2a response against only two antigens, Cha and myosin. Importantly, there was a distinct difference in the IgG2a/IgG1 ratio between the two groups: the average IgG2a/IgG1 ratio was 4.1 in *T. cruzi*–infected mice and 1.1 in HKTC-immunized mice (Table 2). These findings coincide with published reports that *T. cruzi*-infected humans and mice mount a type I immune response [42], [43], [44] and indicate that the HKTC-immunized mice mount a balanced Th1/Th2 autoimmune response.

**Table 2:** The cardiac antigen-specific IgG2a/IgG1 antibody isotype ratio is higher in *T. cruzi*-infected mice than in HKTC-immunized mice.

		*T. cruzi*-Infected Mice					HKTC-Immunized Mice					PBS-Immunized Mice			
	IgG2a		IgG1		G2a∶G1	IgG2a		IgG1		G2a∶G1	IgG2a		IgG1		G2a∶G1
	GMT	Log_2_±SE a	GMT	Log_2_±SE	Ratio	GMT	Log_2_±SE	GMT	Log_2_±SE	Ratio	GMT	Log_2_±SE	GMT	Log_2_±SE	Ratio
**actin**	174.1	7.4±0.2^a^	126.0	7.0±0.2^a^	**1.1**	69.6	6.1±1.8^a^	51.7	5.7±0.6^a^	**1.1**	1.0	0.0±0.8	2.2	1.1±1.2	**--**
**Cha**	696.4	9.4±0.2^a^	2.2	1.1±1.1^a^	**8.5** b	253.9	8.0±0.4^a^	21.6	4.4±0.4^a^	**1.8** b	2.2	1.1±1.2	1.0	0.0±0.8	**--**
**desmin**	7351.7	12.8±0.2^a^	4.6	2.2±1.4^a^	**5.8** b	200.1	7.6±1.3^a^	119.4	6.9±0.8^a^	**1.1**	1.0	0.0±0.8	1.0	0.0±0.8	**--**
**laminin**	919.0	9.8±0.2^a^	5079.7	12.3±0.8^a^	**0.8** b	17.6	4.1±1.3^a^	39.8	5.3±0.8^a^	**0.8**	2.2	1.1±1.2	1.0	0.0±0.8	**--**
**myoglobin**	174.1	7.4±0.2^a^	112.2	6.8±0.2^a^	**1.1** b	95.1	6.6±0.2^a^	143.1	7.2±0.9^a^	**0.9**	1.0	0.0±0.8	2.2	1.1±1.2	**--**
**myosin**	3687.5	11.8±0.2^a^	712.7	9.5±0.3^a^	**1.2** b	517.8	9.0±0.2^a^	159.0	7.3±0.5^a^	**1.2** b	2.2	1.1±1.2	2.2	1.1±1.2	**1.0**
**tropomyosin**	4222.4	12.0±0.5^a^	141.4	7.1±0.2^a^	**1.7** b	293.1	8.2±0.2^a^	263.3	8.0±0.7^a^	**1.0**	1.0	0.0±0.8	1.0	0.0±0.8	**--**
**HKTC**	12800.0	13.6±0.0^a^	2.2	1.1±1.1^a^	**12.4** b	3665.8	11.8±0.9^a^	7311.9	12.8±0.8^a^	**0.9**	4.6	2.2±1.5	4.6	2.2±1.5	**1.0**
**Mean**					**4.1**					**1.1**					

All analyses were conducted at 21 days post-infection. n = 5 for each group except n = 3 for PBS-immunized group. GMT, geometric mean titer.

a *P*<0.05 compared to the PBS/CFA-immunized control group.

b *P*<0.05 for the IgG2a response compared to the corresponding IgG1 response for each cohort.

### *T. cruzi*-infected and HKTC-immunized mice develop cellular autoimmunity

**Figure 3:**
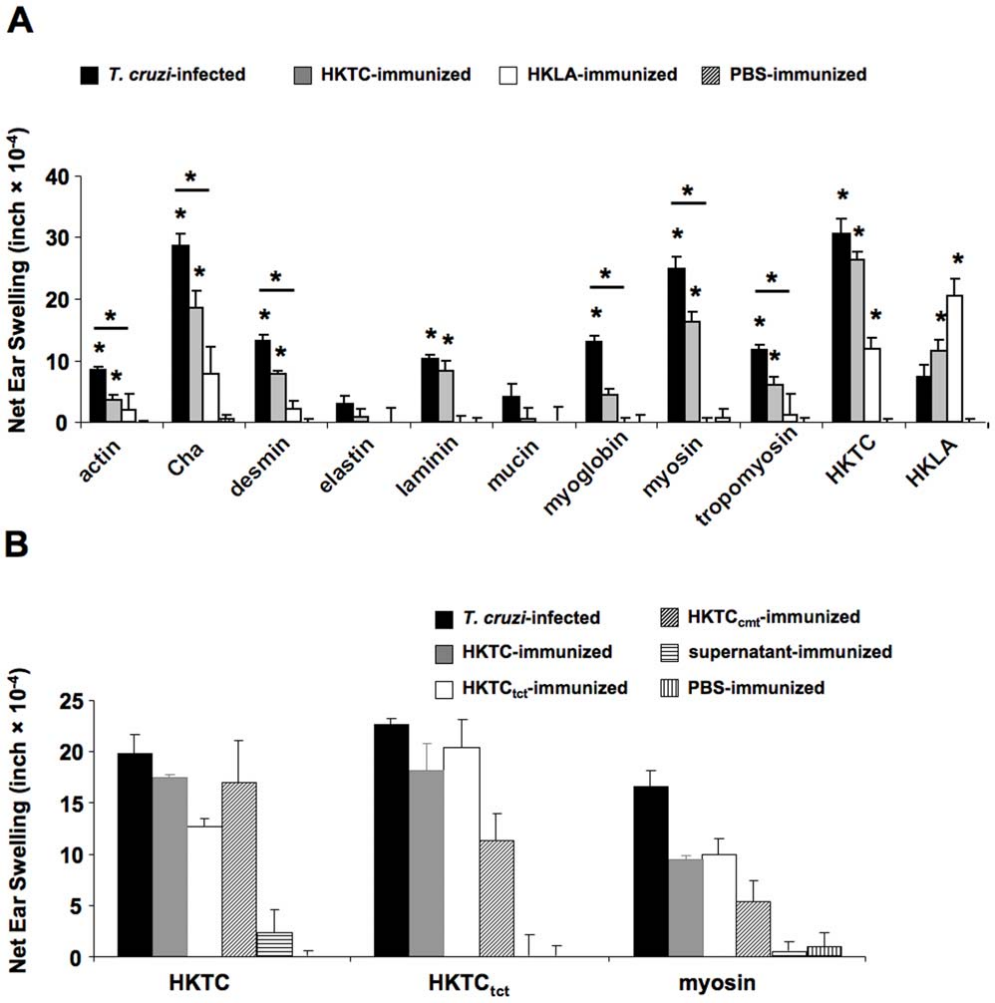
*T. cruzi*-infected and HKTC-immunized mice develop DTH specific for a number of cardiac and non-cardiac proteins. A/J mice were infected with *T. cruzi*, immunized with HKTC, immunized with HKLA, or injected with PBS. (A) At 21 d.p.i., antigen-specific DTH responses to a panel of cardiac and non-cardiac antigens were measured by a standard 24-hour ear swelling assay. (B) DTH responses specific for HKTC and myosin were measured in A/J mice immunized heat-killed *T. cruzi* epimatigotes (HKTC), *T. cruzi* tissue-culture trypomastigotes (HKTC_tct_) *T. cruzi* cultured metacyclic trypomastigotes (HKTC_cmt_), supernatant from *T. cruzi-*infected myoblast cultures (supernatant), or injected with PBS. Data represent the net ear swelling response over the basal response to an irrelevant antigen (BSA). Error bars indicate SEM (n = 4). Data are representative of two independent experiments. * *P*<0.05 compared to the PBS control group.

We next investigated whether *T. cruzi*-infected and HKTC-immunized mice develop cellular autoimmunity to a similar panel of antigens by assessing DTH responses, a well-established and sensitive measure of *in vivo* cellular immunity. Both infected and immunized mice developed significant DTH responses to most of the heart-abundant antigens, but not the non-cardiac antigens (Fig. 3A). However, for all of the autoantigens, the response in infected mice was significantly higher than the corresponding response in HKTC-immunized mice. This suggests that infected mice mount a more robust cell-mediated autoimmune response than mice immunized with HKTC, while there is no statistically significant difference in the anti-parasite DTH responses between the two groups. As expected, HKLA-immunized mice only developed significant DTH responses to parasite proteins but not against any of the autoantigens tested (Fig. 3A). There was crossreactivity between HKTC and HKLA in the infected and immunized mice, which is not surprising due to the close relationship between these two protozoans (Fig. 3A). To determine whether immunization with HKTC made from different life-cycle stages of the parasite results in differential induction of myosin-specific and HKTC-specific cellular immune responses, we immunized mice with HKTC_tct_ and HKTC_cmt_, and assessed DTH responses against HKTC, and against cardiac myosin as an indicator of autoantigen-specific responses. There was no significant difference in the magnitude of DTH response to HKTC or cardiac myosin between mice immunized with HKTC, HKTC_tct_, or HKTC_cmt.;_ although HKTC_cmt_ induced slightly lower DTH responses against HKTC_tct_ and myosin than the other HKTC preparations (Fig. 3B). Overall, these results demonstrate that immunization with *T. cruzi* proteins from epimastigotes, tissue culture-derived trypomastigotes, and culture-derived metacyclic trypomastigotes induce autoreactive cellular immune responses that are of similar antigen specificity but lower magnitude compared to the response that develops in *T. cruzi*-infected mice.

### A higher proportion of splenocytes produce IFN-γ in response to autoantigens and HKTC in *T. cruzi* infected mice than in HKTC-immunized mice

**Figure 4:**
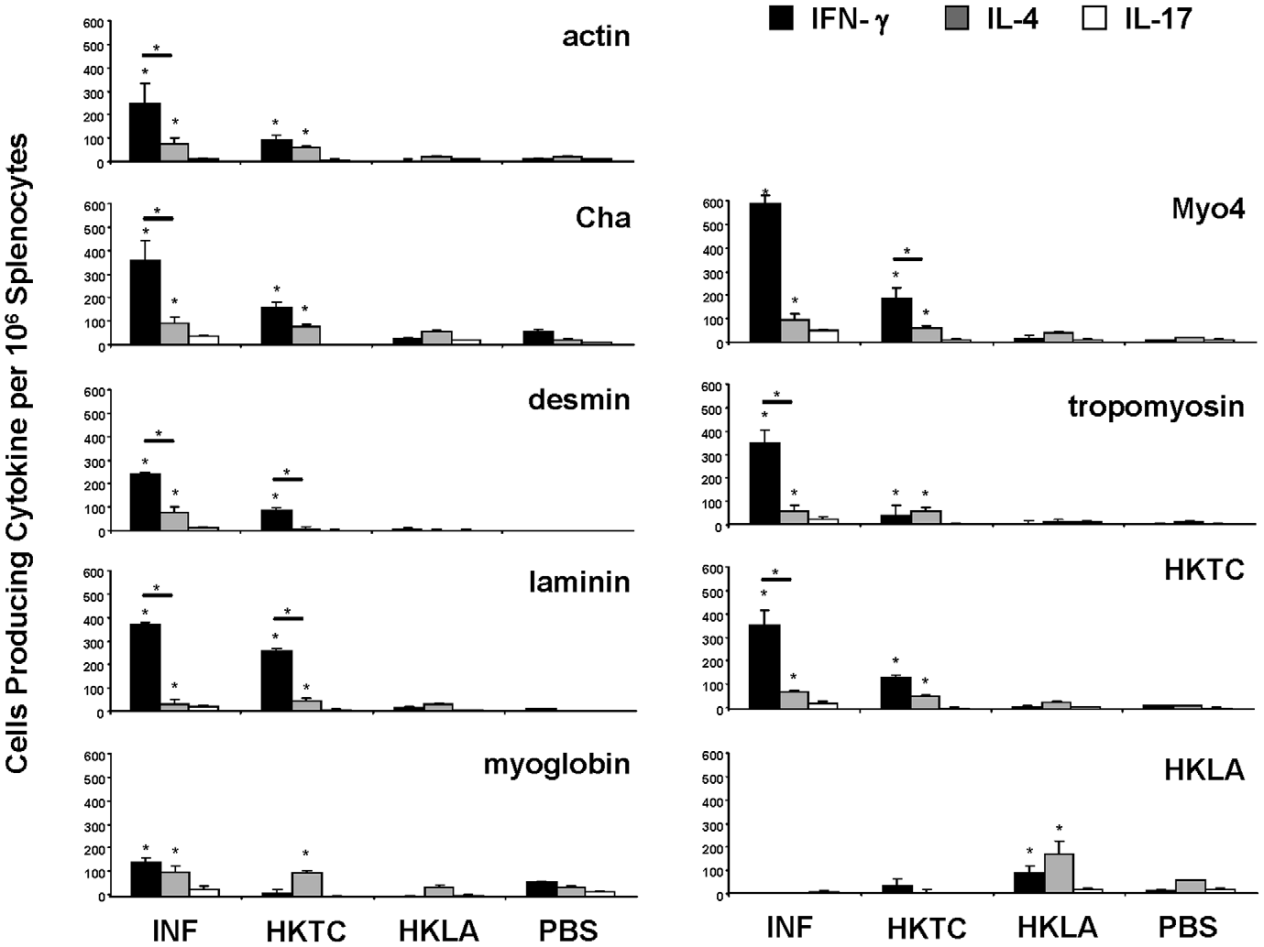
Splenocytes from *T. cruzi*-infected mice produce a different cytokine profile when stimulated with cardiac autoantigens than those from HKTC-immunized mice. A/J mice were infected with *T. cruzi*, or immunized with HKTC, HKLA, or PBS. At 21 d.p.i., splenocytes were isolated and combined from five mice per group and stimulated with the indicated antigens. (A) IFN-γ, (B) IL-4, and (C) IL-17 production were measured by ELISPOT. Data are representative of four independent experiments. Error bars indicate SEM (n = 4). * *P*<0.05 compared to the PBS control group.

We hypothesized that infected mice would produce a more dominant Th1 response than HKTC-immunized mice, based on the observations that infected mice displayed a higher antigen-specific IgG2a/IgG1 ratio. To characterize and compare the relative contributions of Th1, Th2, and Th17 cells, we measured IFN-γ, IL-4, and IL-17, respectively. Due to the *in vitro* cytotoxicity of whole cardiac myosin [23], we stimulated cultured cells with an immunodominant recombinant fragment of cardiac myosin, Myo 4 [23], to assess myosin-specific cytokine responses. Significant numbers of cells producing IFN-γ and IL-4 in response to most or all of the experimental autoantigens were isolated from the spleens of *T. cruzi*-infected and HKTC-immunized mice (Fig. 4). Modest, but statistically significant, numbers of splenocytes producing IL-17 in response to all of the autoantigens tested were detected in infected mice, but no significant IL-17 response was detected in the bulk splenocyte preparation from HKTC-immunized mice (Fig. 4). The frequency of IFN-γ producing cells in infected mice was, on average, 5.2-fold higher than the frequency of cells producing IL-4, and approximately 16-fold higher than the frequency of cells producing IL-17 in response to stimulation with each respective antigen. The frequency of the autoantigen-specific IFN-γ responses in HKTC-immunized mice were, on average, only 2.2-fold higher than the IL-4 responses, with the exception of the desmin-specific response which was approximately 10-fold higher. Most interestingly, the frequency of autoantigen-specific IFN-γ-producing cells in infected mice averaged 4.8-fold higher than in HKTC-immunized mice. The frequency of autoreactive IL-4 producing cells was similar in both groups, with the exception of the desmin-specific response, which was markedly higher in infected mice. HKLA-immunized produced a significant IFN-γ and IL-4 response against HKLA, but not against any other antigens. These observations suggested that, in response to both autoantigens and HKTC, *T. cruzi*–infected mice mount a dominant Th1 response with a lesser Th17 component, whereas the immune response of HKTC-immunized mice is only slightly shifted toward Th1 from Th2 and lacks a significant Th17 component.

### *T. cruzi*-infected mice produce higher frequencies of IFN-γ and IL-17 single positive and IFN-γ^+^ IL-17^+^ double positive T cells than HKTC-immunized mice

**Figure 5:**
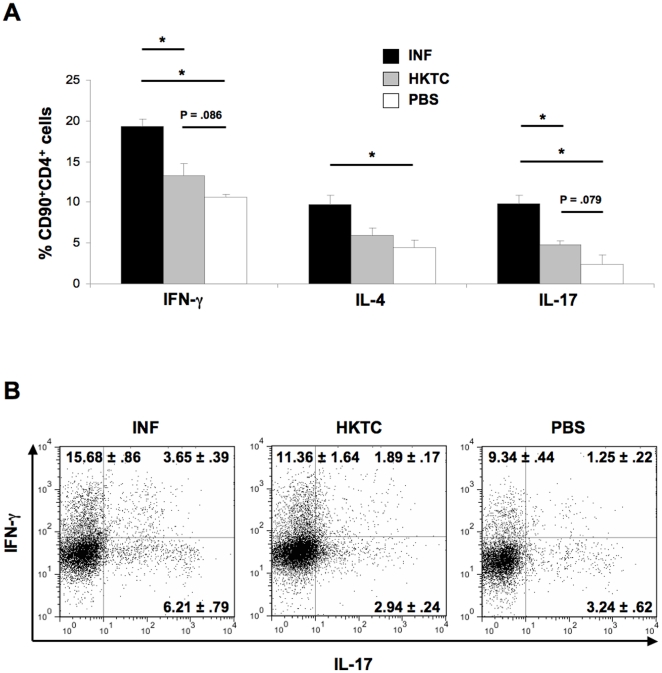
*T. cruzi*-infection but not HKTC immunization induces robust autoreactive Th1 and Th17 responses. A/J mice were infected with *T. cruzi* (INF), or immunized with HKTC (HKTC), or PBS (PBS). At 21 d.p.i., splenocytes were isolated and stimulated with HKTC or Myo 4. Intracellular cytokine staining of CD90+CD4+ splenocytes stimulated with anti-CD3 for IFN-γ, IL-4, and IL-17 was analyzed via flow cytometry. (A) The mean percentage of cytokine-positive cells is shown. Error bars indicate SEM (n = 4 for INF and HKTC; n = 3 for PBS). Data are representative of two independent experiments. (B) Representative plots of the cells quantified in panel A are shown. The mean percentage and SEM of IFN-γ+IL-17-, IFN-γ+IL-17+, and IFN-γ−IL-17+ cells are indicated.

Because both CD4+ T helper cells and other cell types including some CD8+ T effector cells produce IFN-γ and IL-17, we analyzed cytokine production specifically from CD90+CD4+ lymphocytes via flow cytometry to determine the relative contribution of Th1, Th2, and Th17 cells to the observed cytokine response. The percentage of CD90+CD4+ T cells producing IFN-γ, IL-4, and IL-17 when stimulated with a polyclonal T cell activator was significantly increased in *T. cruzi*-infected mice relative to PBS-immunized control mice (Fig. 5A). The percentage of IFN-γ^+^ cells was nearly two-fold greater than the percentage of IL-4^+^ cells, consistent with our earlier findings that suggested *T. cruzi*-infected mice mount a Th1-dominant immune response. HKTC-immunized mice produced slightly increased levels of IFN-γ- and IL-17-producing CD90+CD4+ T cells relative to PBS-immunized controls; however this increase was not statistically significant, (p = .086 and .079, respectively). HKTC-immunized mice also did not develop an increased percentage of IL-4^+^ CD90+CD4+ T cells. The frequency of IFN-γ^+^ and IL-17^+^ cells in HKTC-immunized mice was significantly lower than in *T. cruzi*-infected mice, while there was no significant difference in the levels of IL-4+ cells. We further analyzed the IFN-γ^+^ and IL-17^+^ cells to determine whether these findings were entirely attributable to increased frequencies of IFN-γ and IL-17 single-positive populations, representing traditional Th1 and Th17 cells, respectively, or if *T. cruzi*-infected or HKTC-immunized mice developed increased frequencies of the more recently described IFN-γ^+^IL-17^+^ double positive population. Because increases in the frequencies of IFN-γ^+^IL-17^+^ have been positively correlated with disease severity in several models of inflammatory and autoimmune diseases, we hypothesized that the decreased total numbers of IFN-γ^+^ and IL-17^+^ CD90+CD4+ T cells in HKTC-immunized mice, relative to *T. cruzi*-infected mice, might be partially attributable to a decrease in this IFN-γ+IL-17+ population. We found that *T. cruzi*-infected mice produced significantly increased percentages of IFN-γ+IL-17-, IFN-γ^−^IL-17^+^, and IFN-γ^+^IL-17^+^ cells compared to control mice (Fig. 5B). HKTC-immunized mice produced similar frequencies of IFN-γ^−^IL-17^+^ and IFN-γ^+^IL-17^+^ cells as control mice, and a slight but not significant increase in IFN-γ^+^IL-17^+^ cells (p = .066) (Fig. 5B). The difference in frequencies of IFN-γ^+^IL-17^−^ between HKTC-immunized and *T. cruzi-*infected mice strongly trended towards significance (p = .058), and the difference in frequencies of IFN-γ^−^IL-17^+^ and IFN-γ^+^IL-17^+^ were significant. These results support our hypothesis that HKTC-immunization does not induce the same type (Th1-skewed) of immune response that develops in response to *T. cruzi* infection.

### *T. cruzi*-infected mice have higher serum IL-12 concentrations than HKTC-immunized mice

**Figure 6:**
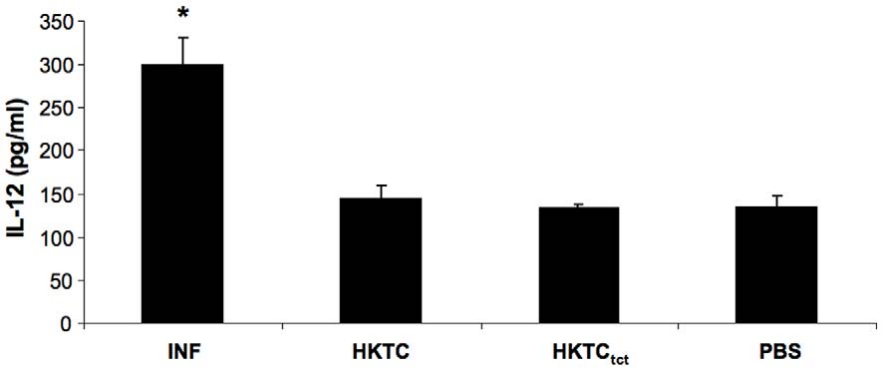
*T. cruzi*-infected mice have higher serum IL-12 than do HKTC-immunized mice. A/J mice were infected with *T. cruzi* (INF), immunized with epimastigote HKTC (HKTC), trypomastigote HKTC (HKTC_tct_) or PBS (PBS). At 21 d.p.i., sera was collected and IL-12 levels were measured via ELISA. The mean concentration of serum IL-12 is shown. Error bars indicate SEM (n = 8 for INF; n = 7 for other groups). * *P*<0.05 compared to the PBS control group.

Because IL-12 is important for promoting differentiation of and IFN-γ production by Th1 cells, we investigated whether the difference in the magnitude of Th1 immune responses between *T. cruzi*-infected and HKTC-immunized mice is associated with a difference in IL-12 levels. Serum was collected from *T. cruzi*-infected, HKTC-immunized mice, and HKTC_tct_-immunized mice at 21 d.p.i., and measured IL-12 concentrations via ELISA. *T. cruzi*-infected mice had serum IL-12 levels that were approximately two-fold higher than mice immunized with HKTC or HKTC_tct_. The serum IL-12 concentrations of the HKTC-immunized mice and HKTC_tct_-immunized mice were not significantly different from PBS-immunized mice (Figure 6).

## Discussion

Previously, we found that immunization with *T. cruzi* protein extract in CFA induced a robust myosin-specific humoral and cellular autoimmune response, similar to that observed in *T. cruzi*-infected mice. However, the *T. cruzi* protein-immunized mice did not develop significant cardiac histopathology nor did they experience early mortality, suggesting that myosin-autoimmunity alone was not sufficient to induce myocarditis. *T. cruzi*-infected mice are known to develop autoimmunity against numerous non-myosin cardiac antigens, but it was not known whether the autoreactive responses elicited by *T. cruzi* immunization extended to any antigens aside from myosin. In the present study we sought to determine whether the autoreactive immunity induced by immunization with whole HKTC extended to non-myosin antigens, and to analyze the autoimmunity induced by HKTC immunization and *T. cruzi* infection to elucidate any qualitative or quantitative differences that may account for the absence of myocarditis. HKTC was initially prepared from *T. cruzi* epimastigotes for most studies due to the comparative ease of generating the large numbers needed for immunization, and to avoid the potential contamination of trypomastigotes preparations with antigens from the rat cardiac myocytes used to propagate these cells. Subsequent analysis demonstrated that the autoimmunity and cardiac damage resulting from HKTC immunization was similar when tissue culture trypomastigotes or culture metacyclic trypomastigotes were used for HKTC production instead of epimastigotes, validating the clinical relevance of the epimastigote HKTC experimentation.

Serum cTnI, a sensitive and specific marker of cardiac damage, is a widely used indicator of cardiac injury. It has been previously validated in a murine model of experimental autoimmune myocarditis, and demonstrates greater utility than creatine kinase or anti-cardiac myosin antibodies [45], [46], [47], [48], [49]. Increased serum cTnI levels positively correlate with increased levels of histopathological changes in the myocardium, and serum markers are a facile means of tracking cardiac injury [47]. *T. cruzi*-infected A/J mice developed a progressive myocarditis that correlates with rising serum cTnI. Interestingly, we found that HKTC immunization also resulted in a statistically significant increase in serum cTnI. Not surprisingly, given the extensive myocarditis and myocyte necrosis present in the hearts of infected mice compared to the lack of inflammatory cell infiltrate and necrosis in the hearts of HKTC-immunized mice, the magnitude of cTnI elevation was lower in the HKTC-immunized animals. However, because elevated serum cTnI is such a sensitive and specific measure of cardiac damage, this strongly suggests that HKTC is initiating sustained cardiac damage in association with induction of polyantigenic autoimmunity, which has not been previously reported [50]. It is also possible that *T. cruzi*-infected mice and HKTC-immunized mice produce autoantibodies against cTnI, which is not unlikely given the observed polyantigenic humoral immune response in these mice. The presence of autoantibodies against troponins in human patients that have experienced myocardial injury has been reported by several groups, and it has been demonstrated that the presence of these antibodies may cause false lower levels of cTnI release, especially when measured by an immunoassay such as that employed in our studies [51]. This may explain why we did not measure higher levels of cTnI in infected and immunized mice, and we will test this possibility in the future. Importantly, this result indicates that parasite antigens alone can initiate cardiac damage leading to subsequent polyantigenic autoimmunity.

The observation that HKTC immunization results in acute myocardial damage and autoimmunity, but not myocarditis, led us to hypothesize that a difference existed in the type or magnitude of cardiac autoimmunity induced by *T. cruzi* infection and HKTC immunization that led to different disease outcomes in the two groups. We determined that both *T. cruzi* infection and HKTC immunization induce cardiac-specific humoral autoreactivity of similar antigen-specificity against the specific proteins in Table 1, with no significant difference in antibody titers specific for any antigen except for myosin, suggesting that the presence of autoimmunity alone does not determine disease outcome. We next analyzed the isotype profiles of the autoantibody responses to determine whether the types of antibodies produced during *T. cruzi* infection differed from those induced by HKTC immunization. We found that the IgG isotype ratio in infected mice was highly skewed toward IgG2a, while the ratio of IgG2a to IgG1 was nearly equivalent in HKTC-immunized mice. This suggested that *T. cruzi*-infected mice produced large amounts of IFN-γ^+^ cells in relation to IL-4^+^ cells, consistent with a Th1-dominant immune response, whereas HKTC-immunized mice mounted a more balanced Th1/Th2 response.

There is controversy over what T cell subtype(s) drives the autoimmune response in experimental CHD. Th1 cells play an essential role in mediating parasite clearance in *T. cruzi*-infected mice by promoting the expansion and functions of CD8+ effector T cells [33]; however, this response also leads to tissue destruction and ongoing release of host antigens. [52]. Th1 cells are known to drive the pathogenesis of a number of autoimmune diseases, so it is likely that Th1 cells contribute to cardiac autoimmunity in CHD [34], [35], [36], [37]. Th17 responses have also been linked to the pathogenesis of a number of inflammatory and autoimmune diseases, including EAM and experimental autoimmune encephalomyelitis (EAE) [23], [36], [37], [38] and, in fact, a combination of both cell subtypes contributes to the inflammation in many of those models. However, there is evidence that IL-17 plays an anti-inflammatory role during *T. cruzi* infection by indirectly downregulating the functions of pro-inflammatory Th1 cells through an IL-12 and T-bet dependent mechanism [53]. Recently, the collective understanding of how Th1 and Th17 cells might play distinct roles in clearing infections and mediating inflammatory conditions has been complicated by the observation of a subpopulation of CD4+ T cells that simultaneously produce both IFN-γ and IL-17 in certain circumstances, such as in mouse models of EAE and in the coronary arteries of human atherosclerotic patients, and increases in IFN-γ^+^IL-17^+^ have been correlated with increased pathology [54], [55], [56].

Measurement of the frequencies of autoreactive IFN-γ^+^, IL-4^+^, and IL-17^+^ splenocytes from *T. cruzi*-infected and HKTC-immunized mice confirmed the hypothesis that infected mice mounted an immune response skewed toward IFN-γ, the prototypic Th1 cytokine. The frequency of autoantigen-specific IFN-γ-producing cells in the spleens of infected mice was significantly higher than the frequency of cells producing IL-4 in response to the same antigens for 6 of 7 antigens. In HKTC-immunized mice, the frequency of autoantigen-specific IFN-γ-producing cells was significantly higher than the corresponding frequency of IL-4 producing cells only for 3 of 7 antigens, and for 2 of 7 antigens the frequency of IL-4 producing cells was higher. *T. cruzi*-infected mice also generated a significant amount of autoreactive IL-17 positive cells specific for all 7 autoantigens tested, but a significant number of IL-17^+^ cells were not detected in the HKTC-immunized mice in response to these antigens. This suggested that infected mice produced a dominant Th1 response, but HKTC-immunized mice did not, which might account for the lack of myocarditis in HKTC-immunized mice. These results also correlated with the observation that *T. cruzi*-infected mice developed more robust antigen-specific DTH responses than HKTC-immunized mice, reflecting the overall trend than infected mice have a more highly-activated T cell compartment than HKTC-immunized mice.

To specifically assess the relative contribution of Th1, Th2, and Th17 cells to the observed immunity, CD90+CD4+ splenic T helper cells were analyzed via flow cytometry for production of IFN-γ, IL-4, and IL-17, respectively. We found significantly increased percentages of Th1, Th2, and Th17 cells in infected mice relative to PBS-immunized control mice, reflective of a global expansion of Th cells in response to active *T. cruzi* infection. Th1 cells predominated and were nearly twice as abundant as Th2 cells, consistent with our hypothesis and previous reports that *T. cruzi*-infection induces robust Th1 immunity. Although Th1-dominace was verified, the ratio of Th1 to Th2 cells was somewhat decreased compared to the ratio of IFN-γ^+^ to IL-4^+^ antigen-specific bulk splenocytes, most likely due to the contribution of other effector cells such as CD8+ cytotoxic T cells to the total IFN-γ response. A significant Th17 response in infected mice became apparent when analysis was restricted to CD90+CD4+ T cells instead of bulk splenocytes, likely because the greatest proportion of IL-17^+^ cells were CD4+ Th17 cells. Although there was a trend towards significance in the frequency of Th1 and Th17 cells in HKTC-immunized mice relative to control mice, this increase was not significant, and there was also no significant increase in Th2 responses, despite the detection of significant levels of IFN-γ^+^ and IL-4^+^ antigen-specific bulk splenocytes in mice immunized with HKTC. These ambiguous results may reflect the contribution of other cell types to the production of these cytokines.

IL-12 is a cytokine known for its important role in driving Th1-differentiation of naïve CD4+ T cells and promoting IFNγ-production of CD4+ T cells [57], [58]. We proposed that the magnitude of the IFNγ response in *T. cruzi*-infected and HKTC-immunized mice correlated to a difference IL-12 production, and indeed we found that *T. cruzi*-infected mice had IL-12 serum concentrations that were approximately two-fold higher than mice immunized with HKTC or HKTC_tct_. Further experimentation will be necessary to link this association to causation regarding the role of IL-12 in the pathogenesis of CHD, and to assess the kinetics of IL-12 production in *T. cruzi*-infected and HKTC-immunized mice.

Separate from the question of whether *T. cruzi*-induced autoimmunity is necessary or sufficient to induce myocarditis is the question of how autoimmunity develops as a result of exposure to *T. cruzi* antigens. The observation that acute cardiac injury and cardiac autoimmunity develop in response to HKTC immunization in the absence of live parasites suggests that molecular mimicry likely initiates exposure to cardiac antigens, thus promoting the development of cardiac specific immune responses during *T. cruzi* infection [27], [59], [60], [61], [62]. Several specific *T. cruzi* proteins have already been identified as mimics of mammalian proteins, including B13 [60] and cruzipain [4] which are mimics of cardiac myosin, and *T. cruzi* ribosomal P proteins which mimic β1 adrenoreceptors [63]. To evaluate the possibility that our HKTC preparation is directly cardiotoxic, leading to cardiac antigen (including cTnI) release and induction of autoimmunity, we assessed HKTC toxicity to cultured myoblasts *in vitro* by measuring mitochondrial reductase activity via an MTT assay as a surrogate of viability (data not shown). We found that neither HKTC nor HKLA caused significant loss of viability, which suggests, along with the temporal kinetics of the response, that myocardial damage in HKTC-immunized mice is mediated by an immune mechanism such as molecular mimicry, rather than the result of direct HKTC cellular toxicity.

Many of the aforementioned experiments were conducted using HKTC made from *T. cruzi* epimastigotes, which is the life cycle form of the parasite that replicates inside the insect vector, due to the relative ease of propagating this life cycle stage, and to avoid concerns of potential contamination by antigens from the myocytes used to propagate trypomastigotes. Since epimastigotes do not cause disease, we compared the cell-mediated and humoral immunity, and myocarditis that develops following immunization with HKTC made from other, more clinically relevant life cycle stages. No significant differences in the induction of *T. cruzi*-specific or autoantigen-specific DTH or antibody responses were observed in mice immunized with HKTC made from epimastigotes HKTC, tissue culture-derived trypomastigotes (surrogate for bloodform trypomastigotes), or *in vitro* culture-derived metacyclic trypomastigotes. Similar to epimastigote HKTC, HKTC made from the other two life cycle stages did not induce cardiac inflammation or myocyte necrosis. This suggests that our observations are attributable to autoimmunity initiated by antigens conserved among these three life cycle stages of *T. cruzi*.

In order for HKTC immunization to result in autoimmunity involving all seven of the autoantigens described in this paper, one of the following scenarios likely occurs: (i) a number of independent molecular mimicry events occurred involving epitopes of *T. cruzi* antigens initiating cross-reactive responses against one or more epitopes found in each of these autoantigens, or (ii) molecular mimicry occurs between one or a few *T. cruzi* antigens and cardiac proteins and the specificity of the autoimmune response expands via epitope spreading. Given that molecular mimicry is a relatively uncommon and restricted event, we favor the latter explanation. Epitope spreading, which occurs when an initial immune response targeting one antigen causes tissue damage precipitating the release of other self-antigens and generating a cascade of additional, non-cross-reactive autoimmune responses, has already been implicated in the pathogenesis of a number of autoimmune diseases [64], [65], [66], [67] and may be involved in propagating the autoimmunity characteristic of CHD, irrespective of the initial cause. We hypothesize that molecular mimicry does occurs between a limited number of *T. cruzi* antigens and structurally related host proteins, initiating immune responses that result in cardiac damage, as evidenced by troponin release, and that epitope spreading then broadens the spectrum of autoimmunity. These mechanisms occur in concert with other immune mechanisms to contribute to the overall autoimmunity and pathogenesis of CHD, as described in recent reviews [1], [7], [68]. We are currently exploring novel molecular mimicry candidates and further investigating epitope spreading in experimental CHD.

An increasingly popular hypothesis suggests that microbial infections, by inducing production of a milieu of proinflammatory cytokines and other factors, can induce autoreactive T cells to become “autoaggressive” effectors and exact pathological damage [69]. In several models of pathogen-associated autoimmune diseases, adjuvants such as CFA appear capable of recapitulating this inflammatory environment and driving autoreactive T cells to become “autoaggressive,” but in other cases more complex stimuli such as the coadministration of IL-1 and TNF-α were required to achieve this effect [69], [70]. It is possible that for *T. cruzi*–induced autoimmunity to cause cardiac pathology, parasite antigens must be presented in a complex inflammatory environment such as that which develops during active infection of the heart, which is not well recapitulated by the addition of adjuvant in the peripheral subcutaneous depot.

This study unifies the characterization of polyantigenic cardiac autoimmune responses following *T. cruzi* infection and exposure to heat-killed parasites. In both scenarios acute cardiac damage occurs, but only active infection resulted in the development of myocarditis. We have demonstrated that the quality of the immune response is different, especially when the immunoglobulin subclasses and cytokine profiles are compared, even if the quantitative measures of autoimmunity are similar. We conclude that the specific immunological differences resulting from an active infection versus an immunization are critical to development of myocarditis, and that autoimmunity resulting from heat-killed *T. cruzi* immunization is insufficient to induce myocarditis in A/J mice. We propose that a dominant Th1 response may be critical to the immune response generated during *T. cruzi* infection that is essential for tissue inflammation.

## Materials and Methods

### Experimental animals, infections and immunizations

Male A/J mice (Jackson Laboratories, Bar Harbor, ME) were 4 to 6 wk of age at the time experiments were initiated. Mice were anesthetized by a single intraperitoneal injection of avertin, 240 mg/kg of body weight for each experimental manipulation. A cardiotropic substrain of the Brazil strain of *T. cruzi* (Brazil Heart) generated in our laboratory [71] was propagated in H9C2 rat myoblasts to generate trypomastigotes for infection and for production of HKTC_tct_. Mice were infected by intraperitoneal injection of 1×10^4^ Brazil heart strain trypomastigotes in Dulbecco's phosphate-buffered saline (PBS; GibcoBRL, Grand Island, NY) or injected with PBS alone (controls). *Leishmania amazonensis* promastigotes (clone LV 78) were maintained in medium 199 (Gibco BRL, Grand Island, N.Y.) supplemented with 10% heat-inactivated fetal bovine serum (Gibco BRL) and grown at 25°C. To produce HKTC_cmt_, epimastigotes were transformed into metacyclic trypomastigotes via *in vitro* culture in artificial triatomine urine medium (TAU) (190 mM-NaC1, 17 mM-KCl, 2 mM-MgCl, 2 mM-CaC12, 8 mM-potassium phosphate buffer, pH 6.0) for 2 h, then in TAU supplemented with 10 mM glucose, 10 mM proline, 2 mM sodium aspartate, and 50 mM sodium glutamate at 27°C as previously described [72]. For immunizations, mice were immunized with HKTC, heat-killed *L. amazonensis* (HKLA), or PBS in an emulsion of CFA in a 1∶1 ratio. A total volume of 150 µl was distributed in three subcutaneous injections in the dorsal flank. Seven days later, mice were given a booster in an identical manner. Infected mice become moribund starting at 17 d.p.i.; 50% must be humanely sacrificed by 21 d.p.i., and 100% by 25 d.p.i., whereas all of the HKTC-immunized and HKLA-immunized mice survive through 60 d.p.i without loss. Mice were housed in pathogen-free animal facilities, and the use and care of mice were in accordance with the guidelines of the Center for Comparative Medicine at Northwestern University.

### Preparation of antigens

Total heart homogenate and cardiac myosin heavy chain were prepared as described previously [5]. HKTC was prepared by washing *T. cruzi* epimastigotes, tissue culture trypomastigotes, or cultured metacyclic trypomastigotes three times in PBS, resuspending in PBS at a concentration of 6.67×10^8^ parasites/ml unless otherwise noted, and incubating in an 80°C water bath for 10 min. Complete loss of viability was verified by culture of treated parasites for 30 days in both trypomastigote and epimastigote culture medium (DMEM and a liver digest-neutralized, tryptose medium, LDNT). HKLA was prepared using promastigotes in a similar manner. In both HKTC and HKLA whole parasite bodies were observed to be uniformly intact via light microscopy. Parasite preparations at 1×10^7^ parasites/ml were stored at −20°C until use. Englebreth-Holm-Swarm murine sarcoma laminin, rabbit skeletal muscle actin, porcine skeletal muscle tropomyosin, elastin from mouse lung, mucin from porcine stomach, and horse heart myoglobin were purchased from Sigma-Aldrich (Atlanta, GA). Lyophilized proteins were resuspended at 1 mg/ml in sterile PBS. Recombinant human Cha antigen was produced in *Escherichia coli* DH5α strain transformed with the pGEX4T3-Cha plasmid (generously provided by Manuel Fresno, Universidad Autónoma de Madrid, Spain) [28]. Recombinant mouse desmin was produced in *Escherichia coli* DH5α strain transformed with the pKK25-pLysE plasmid. Recombinant Cha and desmin were engineered with GST and hexahistidine tags, respectively, and purified from *E. coli* expressors by affinity chromatography. Purified recombinant proteins were resuspended in sterile PBS at 1 mg/ml. For *in vitro* T cell stimulation, cardiac myosin was substituted with an immunodominant portion of cardiac myosin named Myo 4, which was produced recombinantly as previously described and resuspended in PBS at 5 mg/ml [23].

### Histopathology

Hearts were removed, washed free of blood, dried, weighed and fixed for at least 24 hr in 10% buffered formalin. Fixed hearts were embedded in paraffin, sectioned, stained with hematoxylin and eosin, and examined by light microscopy. Each section was examined for evidence of mononuclear and polymorphonuclear cellular infiltration and was assigned a score between 0 (no involvement) and 4 (100% involvement), with 1, 2, and 3 representing 25, 50, and 75% involvement of the histologic section as previously described [25], [73]. Six sections per heart were analyzed at 20× to determine the histopathological score.

### cTnI ELISA

Serum cTnI levels in *T. cruzi*-infected (n = 4), HKTC-immunized (n = 6), and PBS-immunized (n = 4) mice were measured using a high sensitivity mouse cTnI ELISA kit per manufacturer's instructions. (Life Diagnostics, Inc., West Chester, PA). Mouse sera were collected at −1, 7, 14, and 21 d.p.i. and immediately stored at −80°C until use, then thawed on ice before analysis. Serum samples (50 µl) were diluted 1∶1 with sterile PBS. All samples were analyzed in duplicate. Absorbance was measured at 450 nm in a Kinetic MicroPlate Reader (Molecular Devices, Sunnyvale, CA). Detection limits were based on the linear standard curve obtained with the cTnI standards provided in the kit.

### IgG and IL-12 ELISA

Approximately 200 µl sera was collected from retro-orbital bleed prior to sacrifice at 21d.p.i. and immediately stored at −20°C until use, then thawed on ice before analysis. Levels of antigen-specific total immunoglobulin G (IgG), IgG1, and IgG2a were measured by ELISA as described previously [5]. IgG antibody titers were defined as the highest serum dilution that produced an absorbance value at optical density of 450 nm that was at least 2 standard deviations above the mean absorbance for negative control samples (pooled sera from uninfected mice, n = 10) included on each plate. Levels of IL-12 were measured per manufacturer's instructions (Ebioscience, San Diego, CA). All samples were analyzed in duplicate. Absorbance was measured at 450 nm in a Kinetic MicroPlate Reader (Molecular Devices, Sunnyvale, CA). Detection limits were based on the linear standard curve obtained with the IL-12p70 standards provided in the kit.

### Measurement of delayed-type hypersensitivity

Antigen-specific delayed-type hypersensitivity (DTH) was quantified using a standard ear swelling assay [5]. Antigen-induced ear swelling was the result of mononuclear cell infiltration and exhibited typical DTH kinetics (*i.e.*, minimal swelling at 4 hr and maximal swelling at 24 hr to 48 hr post injection). Net ear swelling was determined by subtracting the mean swelling generated in response to an irrelevant antigen (BSA) from the mean response generated by the test antigen, as measured with a Mitutoyo model 7326 engineer's micrometer (Mitutoyo MTI Corporation, Aurora, Ill.).

### ELISPOT assay

RBC-free single cell suspensions were prepared from mouse spleens as described previously [23]. The cells were washed and resuspended in complete DMEM (supplemented with 50 µM β-mercaptoethanol, 10% fetal bovine serum, 2 mM L-glutamine, 0.1 M nonessential amino acids, and 100 U/ml penicillin). Cells were plated at 5×10^5^ cells per well and cultured in 96-well plates that were precoated with 2 µg/ml anti-IFN-γ, anti-IL-4, or anti-IL-17 (BD Biosciences, San Diego, CA, USA) in PBS. Cells were stimulated with 125 µg/ml of actin, cardiac myosin (a polypeptide named Myo 4 spanning amino acids 1074–1646) [23], Cha antigen, desmin, laminin, myoglobin, tropomyosin, HKTC, or BSA (negative control) and incubated at 37°C in 5% CO_2_ for 36 h. Plates were washed to remove cells and incubated with alkaline phosphatase-conjugated anti-biotin antibody for 2 hr and developed using 5-bromo-4-chloro-3indoyl phosphate and p-nitroblue tetrazolium phosphate in 100 mM Tris, 1 mM MgCl_2_ (pH 9.5). Cytokine production was quantified using an Immunospot apparatus (Cellular Technology Ltd, Shaker Heights, OH, USA).

### Intracellular cytokine staining and flow cytometric analysis

RBC-free single cell suspensions were prepared from mouse spleens as described previously [23]. The cells were washed and resuspended in complete DMEM. Cells were plated at 5×10^5^ cells per well and cultured in 96-well plates for 72 hr while being stimulated with anti-CD3 (2c11 hybridoma) or BSA (Sigma-Aldrich). Following stimulation cells were washed and re-stimulated with 5 ng/ml phorbol 12-myristate 13-acetate (Sigma-Aldrich) and 500 ng/ml ionomycin (Fisher Scientific) for 3 h, then with 1∶1000 Golgi Plug and 1∶1000 Golgi Stop (BD Biosciences) for 3 h. Cells were washed and blocked for 10 min with 1∶100 anti-CD16/32 (Ebioscience) then stained with anti-CD90-PE-Cy7 and anti-CD4-PE-Cy5.5 (Ebioscience) for 20 min and washed. Cells were then fixed with 4% paraformaldehyde at 4C for 20 min and permeabilized using the Perm/Wash buffer from the BD Cytofix/Cytoperm Plus Fixation/Permeabilization Kit (BD Biosciences). Intracellular cytokine staining was performed using anti-IFN-γ-FITC, anti-IL-4-APC, and anti-IL-17-PE (BD Biosciences). Flow cytometry was performed using an LSRII flow cytometer with FACS Diva software (Becton Dickinson, Mountain View, CA), with a minimum of 10,000 events recorded, and analyzed with FlowJo 7.6 Software.

### Statistical analysis

DTH values and antibody titers were log_2_-transformed prior to statistical analysis if they were not normally distributed. Titers of 0 were replaced by 1 for logarithmic transformations. For comparison of two groups, significance was determined by Student's *t* test. For comparison of multiple groups and a control, the significance was assessed by one-way analysis of variance, followed by adjustment for multiple comparisons by the Dunnett test (post hoc analysis). The control group for comparison is specified in each figure legend. *P* values of <0.05 were considered significant.

### Ethics Statement

All animal experiments were conducted under protocols approved by the Northwestern University Animal Care and Use Committee (Approval 2006-0059), which is fully accredited by the Association for Assessment and Accreditation of Laboratory Animal Care International (File Number: 000602).” The transportation, care, and use of all animals was conducted in accordance with the Animal Welfare Act (7 U.S.C. 2131 et. seq.) and other applicable Federal laws, guidelines, and policies, including those guidelines set forth by the NIH.
