# Correction: Heat-Killed *Trypanosoma cruzi* Induces Acute Cardiac Damage and Polyantigenic Autoimmunity

**DOI:** 10.1371/annotation/118b702e-9609-4f91-afba-6a76f8f1f14a

**Published:** 2011-02-09

**Authors:** Kevin M. Bonney, Joann M. Taylor, Melvin D. Daniels, Conrad L. Epting, David M. Engman

There is an error in the author contributions. KMB should be listed in "wrote the paper" rather than DME. The correct author contributions are: Conceived and designed the experiments: KMB CLE DME. Performed the experiments: KMB JMT MDD. Analyzed the data: KMB. Contributed reagents/materials/analysis tools: KMB. Wrote the paper: KMB. Edited the manuscript: CLE DME.

